# Imaging the postoperative patient: long-term complications of gastrointestinal surgery

**DOI:** 10.1007/s13244-015-0451-8

**Published:** 2015-12-05

**Authors:** Daniel Ramos-Andrade, Luísa Andrade, Catarina Ruivo, Maria Antónia Portilha, Filipe Caseiro-Alves, Luís Curvo-Semedo

**Affiliations:** Medical Imaging Department, Coimbra Hospital and University Centre, Coimbra, Portugal; Faculty of Medicine, University of Coimbra, Coimbra, Portugal

**Keywords:** Postoperative complications, Intestinal obstruction, Afferent loop syndrome, Abdominal hernia, Surgical adhesions

## Abstract

**Objectives:**

The objectives of this review are (1) to become acquainted with the long-term complications of surgery of the gastrointestinal tract, and (2) to appreciate the appropriate use of imaging in the assessment of long-term complications.

**Background:**

Gastrointestinal tract surgery comprises a group of procedures performed for a variety of both benign and malignant diseases. In the late postoperative setting, adhesions and internal hernias are the most important complications. and they can be further complicated by volvulus and ischemia. At present, computed tomography (CT) is the workhorse for evaluating late postoperative complications. Accurate imaging assessment of patients is essential for adequate treatment planning.

**Imaging findings or procedure details:**

In this pictorial essay we will review the most frequent long-term complications after gastrointestinal surgery, including adhesions, afferent loop syndrome, closed-loop obstruction, strangulated obstruction, internal hernias, external hernias, anastomotic strictures and disease recurrence. Examples will be depicted using iconography from the authors’ imaging department.

**Conclusions:**

Knowledge of the most frequent complications after gastrointestinal surgery in the late postoperative period is of paramount importance for every radiologist, so that potentially life-threatening situations can be promptly diagnosed and adequate therapy can be planned.

**Teaching points:**

• *Long-term postoperative complications of gastrointestinal tract surgery can be divided intoprocedure-relatedanddisease-relatedcategories.*

• *The most commonprocedure-relatedcomplications are internal hernias and adhesions.*

• *The most frequentdisease-relatedcomplications are mainly associated with neoplastic or inflammatory recurrence.*

• *Computed tomography is the most useful examination when such complications are suspected.*

## Introduction

An acquaintance with the types of surgery, including the most common anastomosis, and with their most frequent complications are the first steps in preventing misdiagnosis of potentially life-threatening complications following gastrointestinal surgery.

The radiologist is often faced with altered anatomic findings that hamper the ability to differentiate between an expected postoperative finding and a real complication. Therefore, communication with the referring surgeon is strongly advised in such situations before performing a diagnostic examination.

Gastrointestinal contrast studies are more commonly used to look for immediate postoperative complications, such as intestinal leak or anastomotic dehiscence.

Computed tomography (CT) is currently the workhorse for evaluating late postoperative complications, with the exception of magnetic resonance imaging (MRI) for suspected recurrence of rectal cancer or inflammatory bowel disease.

Late postoperative complications can be classified as procedure- or disease-related [1] (Table 1).

**Table 1:** Long-term postoperative complications of GI tract surgery

Procedure-related:
• Herniation (internal and external) • Adhesions • Afferent loop syndrome • Anastomotic strictures
Disease-related:
• Neoplastic recurrence • Inflammatory bowel disease recurrence

Adhesions and internal hernias are the most important procedure-related complications in the late postoperative period, and can be further complicated by volvulus and ischemia. Anastomotic strictures are also relatively frequent following GI tract surgery.

Disease-related complications are typically related to disease recurrence involving both neoplastic and inflammatory conditions.

## Procedure-related complications

### Internal hernias

Internal hernias (IH) are defined as the protrusion of the viscera through a normal or abnormal peritoneal or mesenteric aperture within the peritoneal cavity [2–4].

These hernias can be either congenital or acquired. The major classifications of internal hernias include paraduodenal (53 %), pericecal (13 %) and transmesenteric hernias (8 %), hernias through the foramen of Winslow (8 %) and intersigmoid hernias (6 %) [2, 4].

Although paraduodenal hernias are classically regarded as the most prevalent, the incidence of transmesenteric hernias (TMH) has been growing given the increased number of operative procedures that involve Roux-en-Y surgery, such as gastric bypass surgery and liver transplants [2, 3]. In fact, IH are almost as frequent a cause of obstruction as adhesions in patients who receive liver transplants with Roux-en-Y anastomosis confection [5].

Clinically, IH can be asymptomatic, or cause nonspecific intermittent abdominal pain or full-fledged strangulated obstruction (the most common presentation). As these hernias are difficult to identify clinically, imaging plays a pivotal role in their evaluation. Herniation of the bowel loops through the defect can be a transient phenomenon that can further confound diagnosis. Clinical or imaging evaluation performed during a symptomatic period is more likely to reveal the abnormality [6].

Transmesenteric hernias are particularly prone to complications (volvulus and strangulation), and symptom onset is usually more acute than in other types of internal hernias [2, 6]. They occur in the adult population who has had abdominal surgery, especially Roux-en-Y surgery, which is now the most frequent type of acquired internal hernia according to a recent study [2, 3, 6]. Transmesenteric internal hernias can be divided into three groups: through a defect in the transverse mesocolon with retrocolic Roux limb (the most common type), through a defect in the small-bowel mesentery at the jejuno-jejunal anastomosis, and posterior to the Roux jejunal loop (Peterson defect) [7, 8].

It is important to differentiate small-bowel obstruction (SBO) due to internal hernia from obstruction due to adhesions, since the former requires emergent surgical treatment [9]. A combination of clinical and imaging criteria can help distinguish between the two. In SBO secondary to adhesions, an abrupt angulation of a bowel segment is more likely to occur. Small bowel obstruction due to IH tends to present much longer after surgery, and a collection of dilated small-bowel loops lying adjacent to the abdominal wall, without overlying omental fat and with central displacement of the adjacent colon, associated with crowding, distortion, and engorgement of mesenteric vessels, is seen on CT [7]. A cluster of loops of small bowel cephalic to the transverse mesocolon between the stomach and spleen in the left upper quadrant is typical of the transmesocolic subtype, and a group of small-bowel loops in a peripheral abdominal location is typical of the transmesenteric jejuno-jejunal type [7, 10]. The Peterson type of TMH has no distinctive findings, but because the herniation most often occurs from right to left, there may also be a cluster of dilated loops in the left upper quadrant [2, 7].

Multi-planar reformation (MPR) oriented to the “intestine-mesentery-directed plane” and vertical to this plane should be performed in order to increase the likelihood of detection of the hernial orifice. This can be identified as an area where the mesentery of the closed loop converges on a given multiplanar view; on the plane vertical to the affected intestine, the hernial orifice shows a round/oval configuration of the converged mesentery [8].

**Fig. 1:**
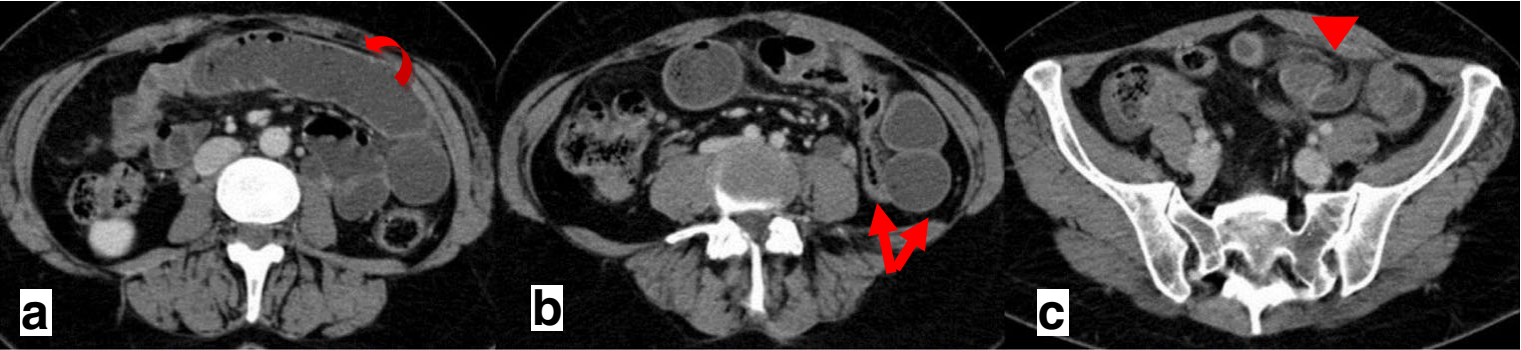
**a**–**c** Surgically-proven TMH in a patient with abdominal pain. There is no fat between the fluid-filled dilated small-bowel loop and the anterior abdominal wall (*curved arrow in a*). The small-bowel loops are placed lateral to the descending colon with medial deviation of it (*arrows in b*). There is twisting of the bowel loops and mesentery—whirl sign (*arrowhead in c*)

Among other signs of internal hernias following Roux-en-Y gastric bypass surgery, which include small-bowel obstruction, clustered small-bowel loops, small bowel other than duodenum located behind the superior mesenteric artery, presence of the jejunal anastomosis to the right of the midline, and engorged mesenteric lymph nodes, the swirling of the mesenteric vessels is most predictive [11] (Fig. 1).

Transmesenteric hernias are generally more difficult than other IH to diagnose by CT because they lack a confining sac and thus are located potentially anywhere within the abdomen [2, 6].

Left-sided paraduodenal IH is the leading differential diagnosis of TMH, but has different distinctive characteristics, such as a sac-like mass of dilated bowel lateral to the ligament of Treitz that displaces and indents the transverse colon and stomach [6].

Volvulus and ischemia of the herniated small bowel are frequent complications of TMH. Typical findings of strangulated closed-loop obstruction are seen (whirl sign, engorged blood vessels, mesenteric edema, ascites and bowel wall thickening) [6].

### Closed-loop bowel obstruction and incarceration

A closed-loop bowel obstruction or incarceration is a type of mechanical intestinal obstruction in which a segment of bowel is occluded at two separate points along its length, almost always adjacent to each other, as the result of a single constrictive lesion. This anatomical configuration can lead to twisting of the loop along its long axis, and thereby produce a bowel volvulus, with subsequent compromise of the vascular supply and ischemia—strangulating obstruction. Although incarceration and strangulation are related phenomena, incarceration may occur without strangulation and may resolve spontaneously [12].

Closed-loop obstruction is most commonly caused by postoperative adhesive bands, and is less frequently due to internal or external hernias [13].

**Fig. 2:**
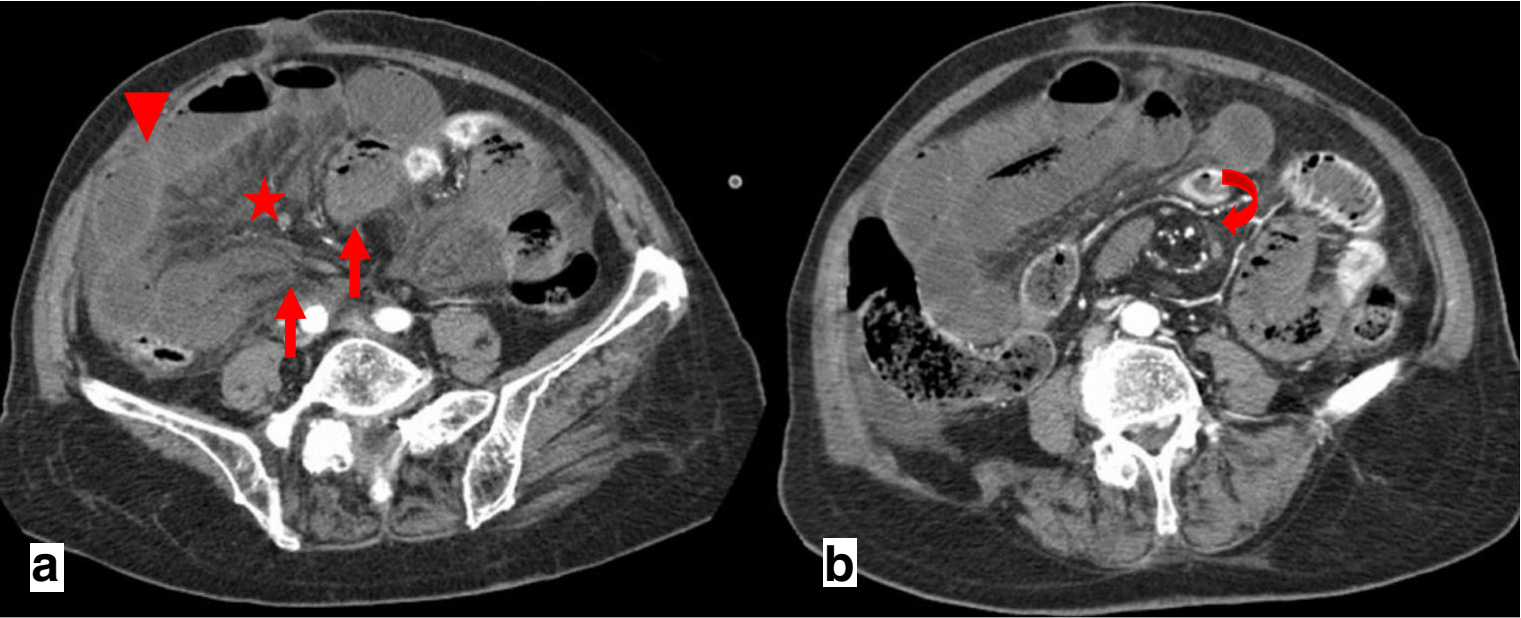
**a**, **b** Patient with abdominal pain because of a closed-loop SBO. Both ends of a fluid-filled distended closed loop taper fusiformly, in a way similar to a beak—the beak sign (*arrows in a*). Mesenteric edema (*star in a*), peritoneal fluid and reduced enhancement of the bowel wall comparatively to other small-bowel loops (*arrowhead in a*) are also evident, findings that suggest strangulation. A slightly cranial image shows the twisting of the mesenteric vessels at the mesenteric root—whirl sign (*curved arrow in b*)
- The whirl sign, which corresponds to the whirled appearance of the mesenteric vessels in the middle of the obstruction point (Fig. 2). The tightness of the whirl pattern reflects the degree to which the mesentery and vessels are rotated. Although hampered by low sensitivity (60 %), a recent study has shown that if this sign is present on a CT scan, a patient is 25 times as likely as a patient without it to undergo surgery [13, 14].
- A radial distribution, which refers to stretched mesenteric vessels converging toward the central point of obstruction (Fig. 3).

CT findings of closed-loop obstruction (incarceration) include the classical sign of mechanical obstruction (a distinct point of transition between dilated and collapsed bowel loops) and additional signs, such as:

- The beak sign, representing the beak-shaped morphology of the dilated loop toward the obstruction, when imaged in a longitudinal orientation (Fig. 2)

**Fig. 3:**
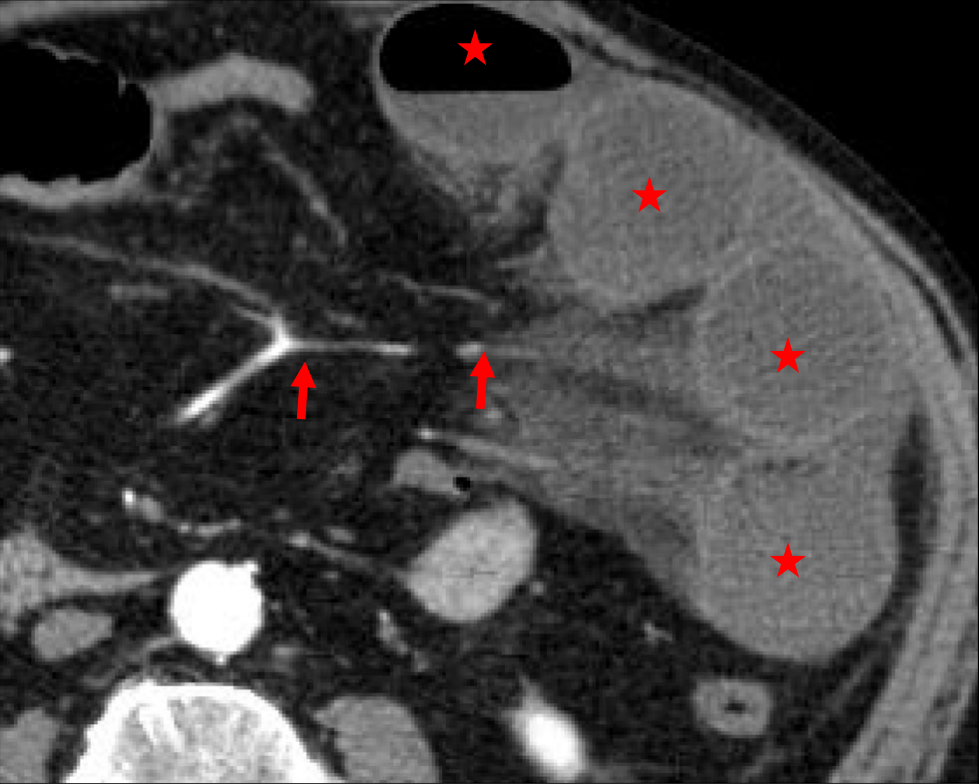
Patient with closed-loop obstruction of the small bowel. Fluid-filled dilated loops of small bowel with a radial distribution (*stars*) and stretching of the mesenteric vessels (*arrows*) are seen
- The U/C sign, which refers to the shape of the incarcerated distended loop (Fig. 4). Although it has been described on axial images when the loop is horizontally oriented, it can also be well depicted on sagittal and coronal reformations when the loop is vertically oriented [13].

**Fig. 4:**
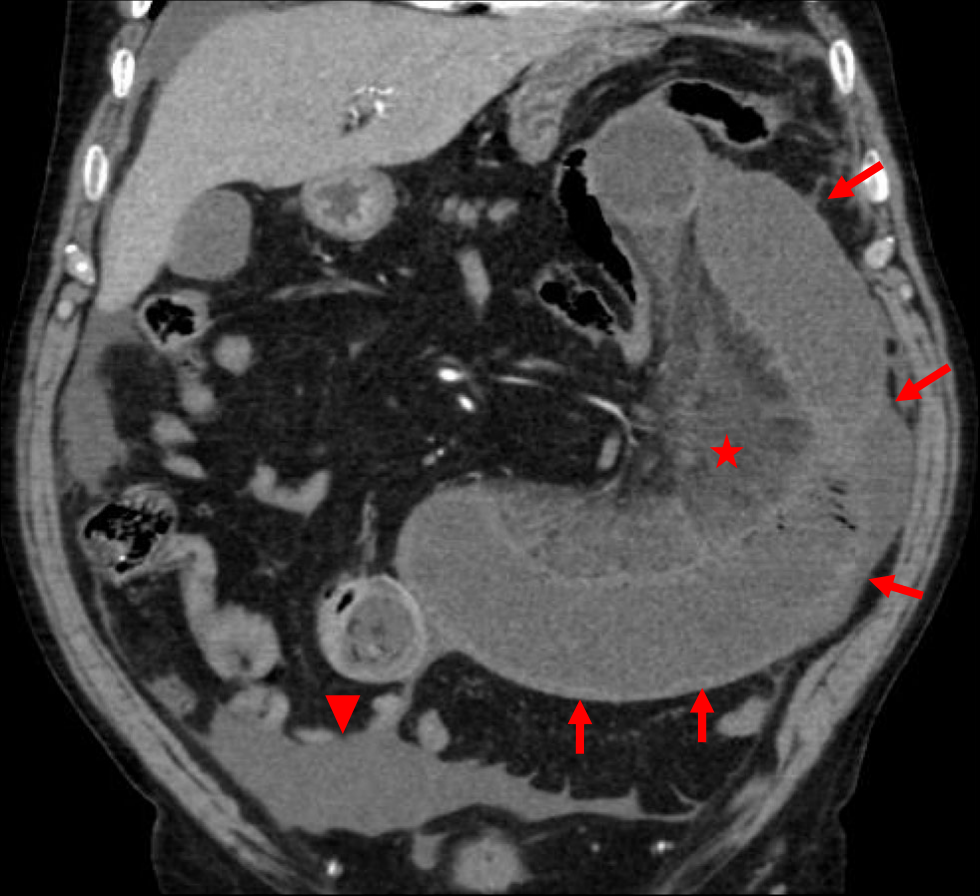
Same patient as Fig. 3. Fluid-filled dilated loop of small bowel with a U/C shape configuration representing a closed-loop obstruction. There is also reduced enhancement of the bowel wall (*arrows*), densification of the mesentery (*star*) and free fluid (*arrowhead*) suggesting strangulation

Multiplanar reformatted images may prove decisive in achieving correct diagnosis of a closed-loop obstruction [13].

CT findings of strangulated obstruction include wall thickening, altered pattern of enhancement of the bowel wall, dilated mesenteric veins, mesenteric edema, ascites, pneumatosis intestinalis, pneumatosis portalis and pneumoperitoneum.

The most common CT finding in bowel ischemia, although nonspecific, is bowel wall thickening. It is caused by mural edema (low attenuation before contrast administration), hemorrhage (high attenuation before contrast administration) and/or superinfection of the ischemic bowel wall. If unenhanced CT is not performed prior to contrast-enhanced CT, the ability to differentiate between intramural hemorrhage and hyperemia and/or hyperperfusion is impaired [15].

On contrast-enhanced CT, a highly specific but less sensitive finding is absent or diminished parietal contrast enhancement. In some cases, prolonged enhancement due to delayed return of the venous blood, with subsequent slowing of the arterial supply, can also be seen [16].

**Fig. 5:**
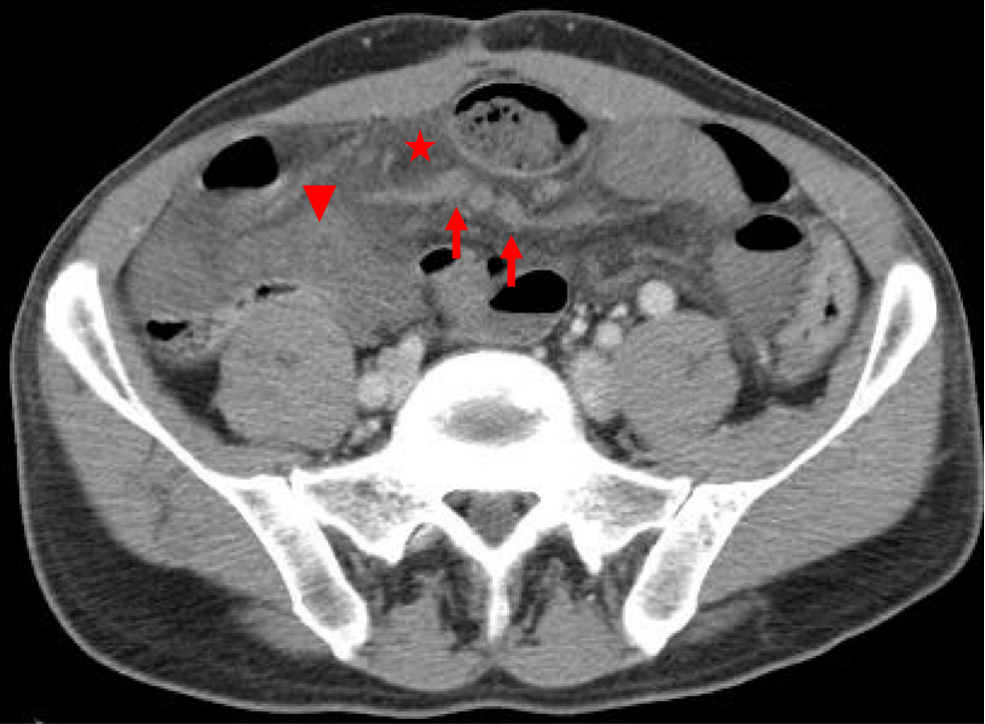
Patient with strangulated obstruction. Engorged mesenteric veins (*arrows*) associated with mesenteric edema (*star*) and free fluid (*arrowhead*) are seen

As ischemia advances, venules in the mesentery become engorged with blood. Mesenteric fat stranding due to edema and/or ascites is also a nonspecific CT finding in acute bowel ischemia (Figs. 4 and 5). In a recent prospective study, fluid in the mesentery adjacent to abnormal (thickened, dilated or both) bowel loops was the individual sign most frequently associated with strangulation (88 %) and had an extremely high negative predictive value [17].

**Fig. 6:**
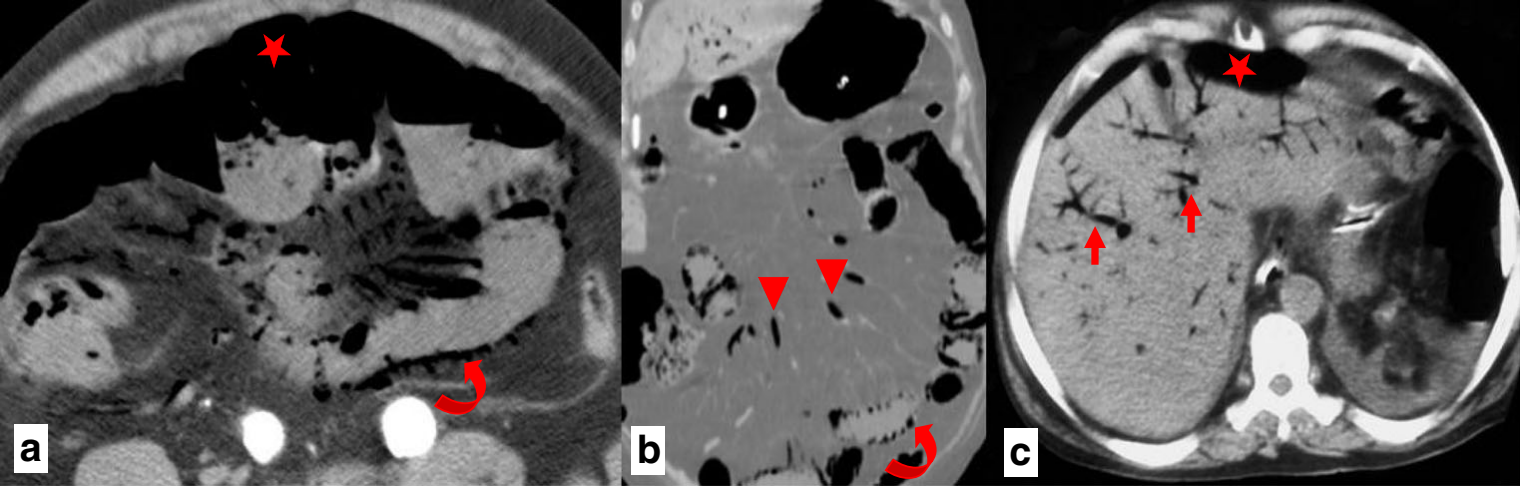
**a**–**c** Patient with bowel ischemia secondary to a strangulated obstruction. Gas inside the mesenteric veins (*arrowheads in b*) coming from a bowel segment that also presents intramural gas (*curved arrows in a, b*) after perforation of a gangrenous bowel wall are seen. Portal venous gas (*arrows in c*) and pneumoperitoneum (*stars in a, c*) are also evident

When bowel wall thinning rather than thickening occurs, transmural small-bowel infarction has probably already ensued. In this setting, it is also more likely for pneumatosis intestinalis, pneumatosis portalis and pneumoperitoneum to develop (Fig. 6). Pneumatosis intestinalis is caused by dissection of luminal gas into the bowel wall across the compromised mucosa. Portomesenteric venous gas is due to the propagation of that gas to the mesenteric venous system, towards the liver. Free intraperitoneal air represents perforation of the gangrenous bowel loop [15, 16, 18].

Clinical and laboratory differentiation between simple obstruction and incarceration/ strangulation is very difficult, and treatment delay is a major prognostic factor for increased morbidity and poor survival. CT is extremely useful in discriminating between these two entities, and it plays an instrumental role in determining appropriate patient treatment (medical versus surgical). Findings of closed-loop obstruction should result in close monitoring, and if clinical symptoms persist, early surgery is suggested. In patients in whom signs of strangulation are already evident, emergency surgery is mandatory [12].

### External hernias

**Fig. 7:**
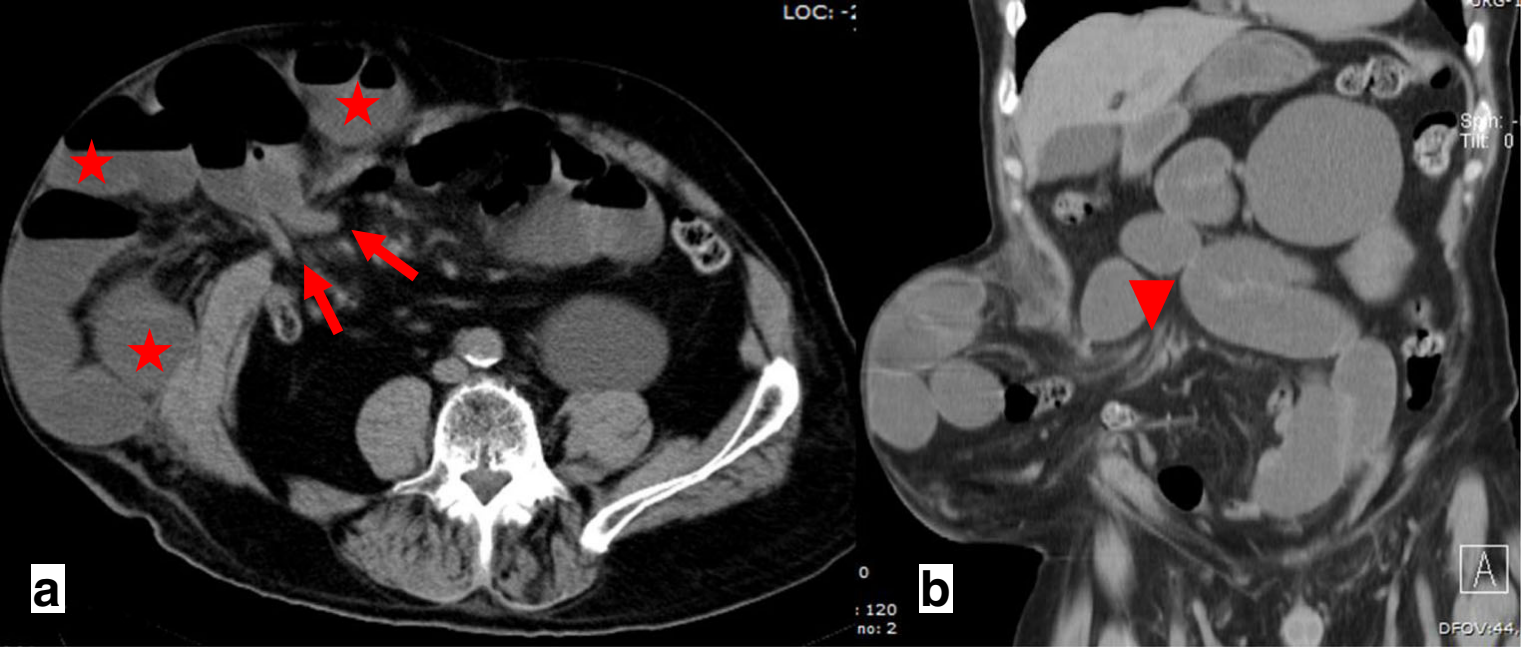
**a**, **b** Patient with an incisional external hernia. A cluster of fluid and air-filled dilated bowel loops is found inside the hernial sac on the right flank (*stars*). Narrowing of the afferent and efferent ends of the closed loop at the site of hernial neck can also be appreciated—the beak sign (*arrows in a*). Stretching of the mesenteric vessels is seen on the coronal reformation (*arrowhead in b*)

**Fig. 8:**
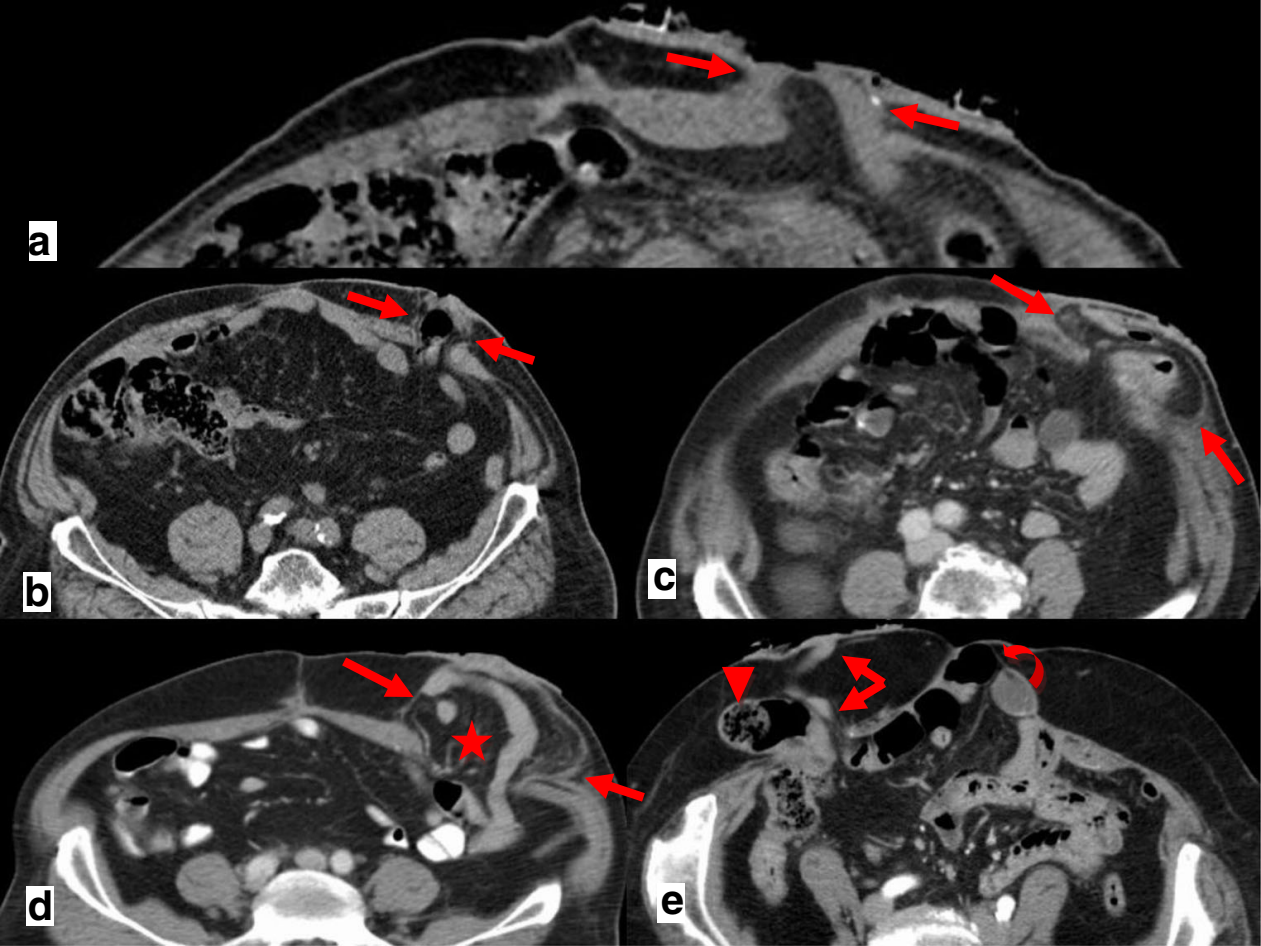
**a** Type 0 “parastomal hernia”: the peritoneum follows the wall of the bowel, with no sac formation (*arrows*). **b** Type Ia “parastomal hernia”: only the bowel forming the colostomy is seen, with a sac <5 cm (*arrows*). **c** Type Ib parastomal hernia: the hernial sac contains only the bowel loop forming the colostomy, but its width is larger than 5 cm (*arrows*). **d** Type II parastomal hernia: there is omentum (*star*) within the hernial sac (*arrows*). **e** Type III parastomal hernia: there are bowel loops (*arrowhead*) other than the one forming the stoma (*arrows*) at the right flank; note also an incisional hernia at the midline in the umbilical region (*curved arrow*)

An incisional hernia is a type of external hernia caused by an incompletely healed surgical wound. Typically, peritoneal fat, with or without incorporation of bowel loops, protrudes through the hernial defect (Figs. 7 and 8e).

Stomal and parastomal hernias are a subtype of incisional hernias that involve herniation of the bowel or the mesentery at the site of or adjacent to a stoma [19]. Even after closure of the enterostomy, the ostomy site can remain a potential area of herniation [20]. Most parastomal hernias occur within 8 months of surgery. The etiology is multifactorial and includes characteristics such as obesity, malnutrition, increased intra-abdominal pressure and chronic respiratory disease [21].

Parastomal hernias are quite common, with an expected prevalence of 33–78 %, depending on clinical or CT evaluation. However, unless obstruction, perforation or stoma malfunction occur, there is no need for surgical repair [19–22].

A recent study suggested a CT classification for parastomal hernias according to the possible contents of the hernial sac, as follows: type I (hernial sac containing the stoma loop), type II (sac containing omentum), type III (sac containing a bowel loop other than stoma) (Table 2). Type 0 and Type Ia are considered normal findings and not true hernias [21] (Fig. 8).

**Table 2:** Parastomal hernia CT classification

Type 0	Peritoneum follows the wall of the bowel, with no sac formation
Type Ia	Only bowel forming the colostomy, with a sac <5 cm
Type Ib	Hernial sac containing only bowel forming the colostomy, with a sac >5 cm
Type II	Hernial sac containing omentum
Type III	Hernial sac containing intestinal loop other than the bowel forming the stoma

**Fig. 9:**
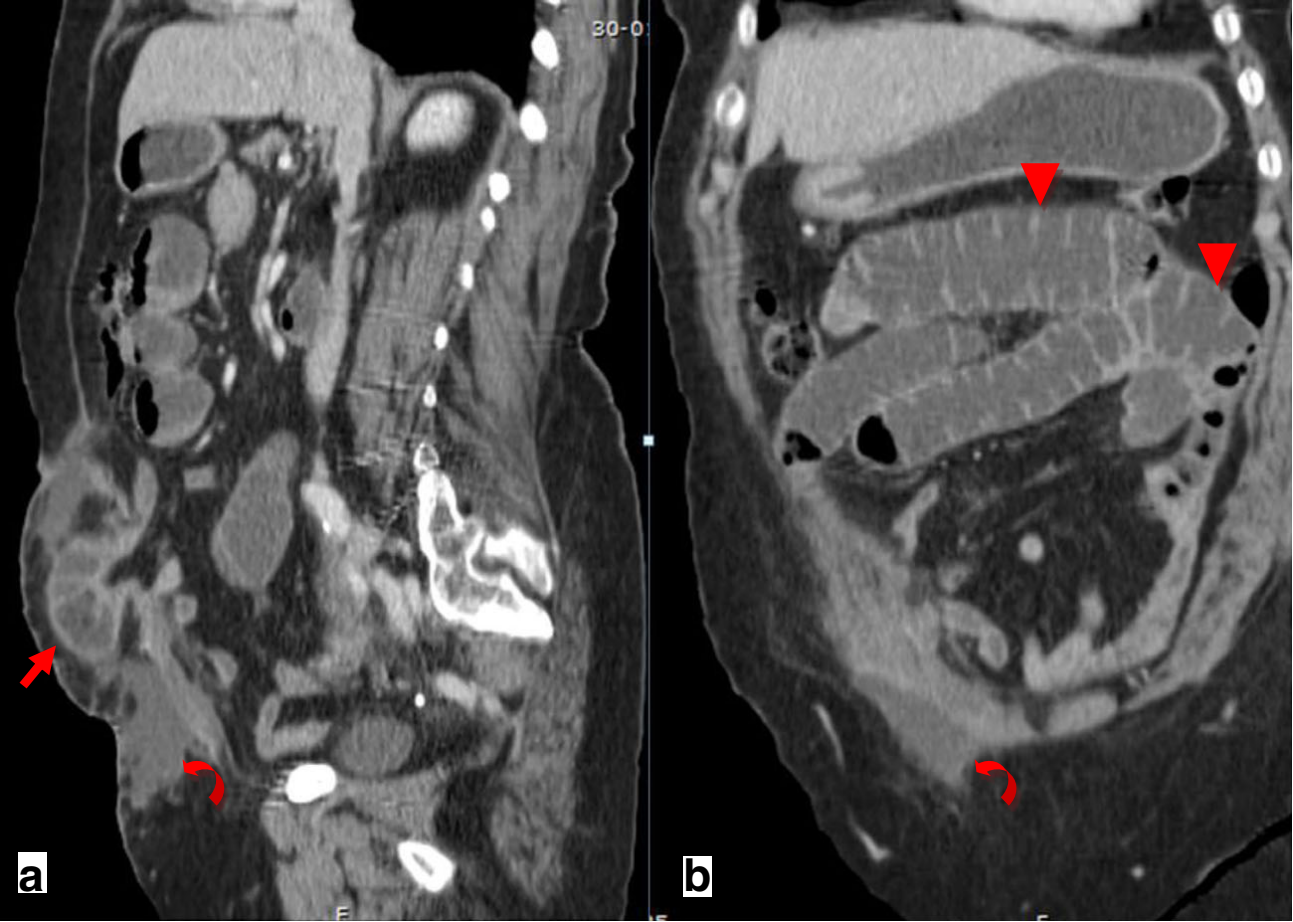
**a**, **b** Abdominal CT of a patient with obstructive symptoms. There is diffuse fluid dilation of small-bowel loops (*arrowheads in b*) proximal to a small segment of dilated small bowel incarcerated at the hernial sac (*arrow in a*). The presence of fat stranding and a moderate amount of free fluid at the site (*curved arrows in a, b*) should raise suspicion of a strangulated hernia

Incarceration and strangulation can supervene with incisional hernias, as with any other abdominal wall hernias elsewhere. Strangulation occurs with typical findings as described above, such as bowel wall thickening, free fluid and fat stranding [19] (Fig. 9).

### Adhesions

Adhesive bands are funiculate structures that form between the peritoneum of tissues and organs, often as a result of injury during surgery. They are composed of fibrous tissue and abundant fat [8].

Adhesions are the most common cause of bowel obstruction after surgery, and they constitute the leading cause of long-term reoperation following abdominal and pelvic surgery.

Bowel loops that pass through the orifice between the adhesive band and the peritoneum form a type of internal hernia that can evolve to closed-loop obstruction. CT findings in this situation are similar to those in other internal hernias (cf. "Internal hernias"). Overt obstructive small-bowel disease related to adhesions can be further subdivided into simple obstruction and closed-loop or strangulated obstruction [23].

Nevertheless, a non-obstructive subtype can also be found. These patients may have intermittent or low-grade small-bowel obstruction, for which CT has poor sensitivity. CT enteroclysis or dynamic MRI may be important options for better demonstrating the transition point [8, 23].

**Fig. 10:**
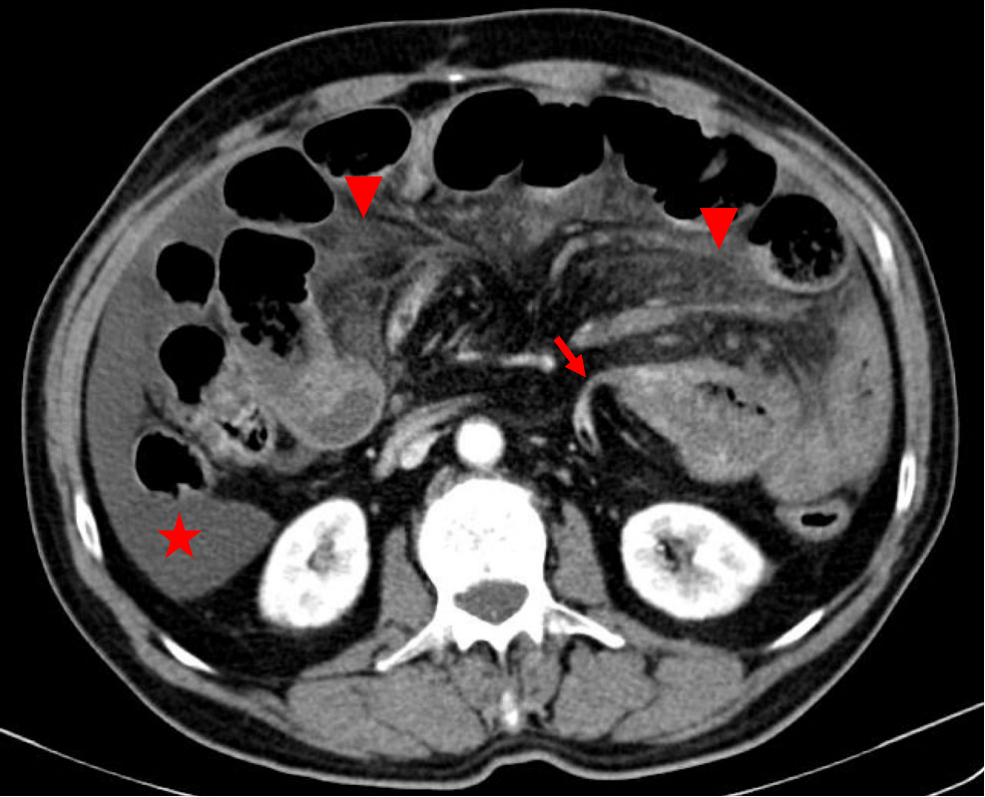
Small-bowel dilation due to an adhesion in a patient with abdominal pain. There is an abrupt narrowing and acute angulation of the loop, with no identifiable underlying lesion (*arrow*). There is also ascites (*star*) and mesentery densification due to edema (*arrowheads*), findings suggestive of strangulated obstruction

**Fig. 11:**
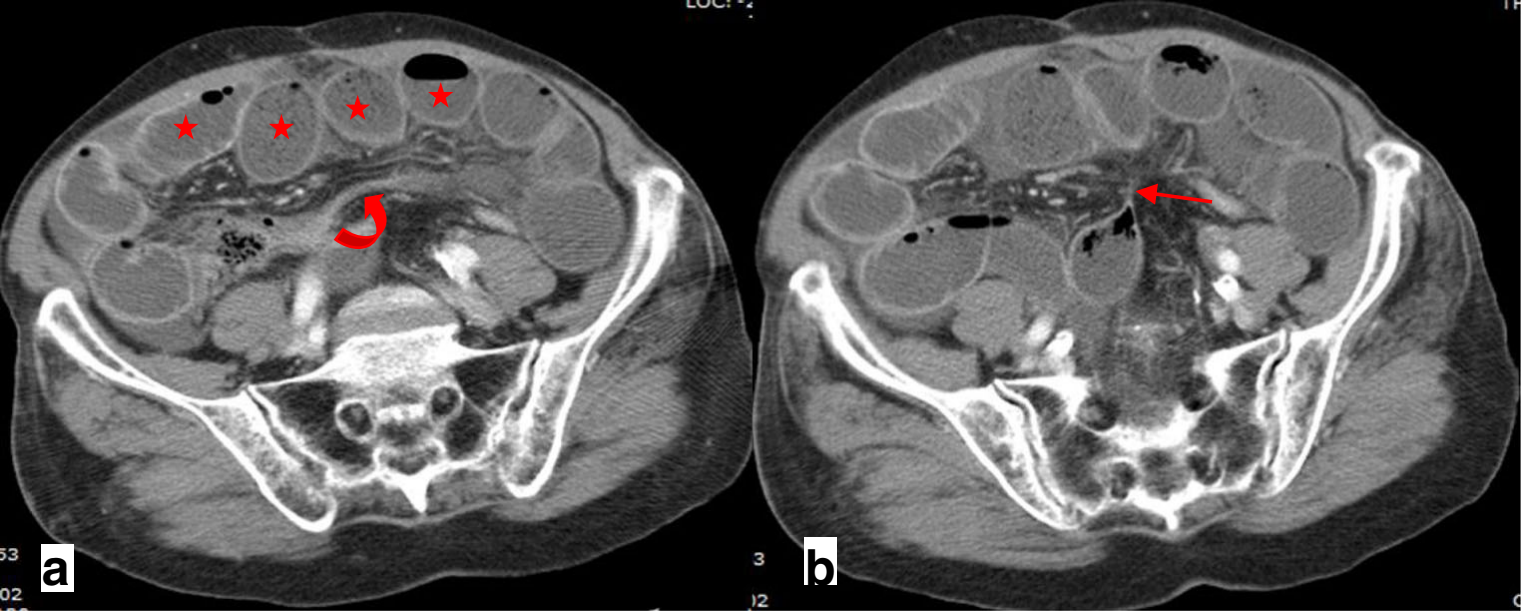
**a**, **b** Adhesion-related SBO in a patient with acute abdominal pain. Dilated small-bowel loops closely applied to the anterior peritoneum (*stars in a*), stretching of the bowel loop (*curved arrow in a*) and traction deformities of the bowel loop (*arrow in b*) are seen

**Fig. 12:**
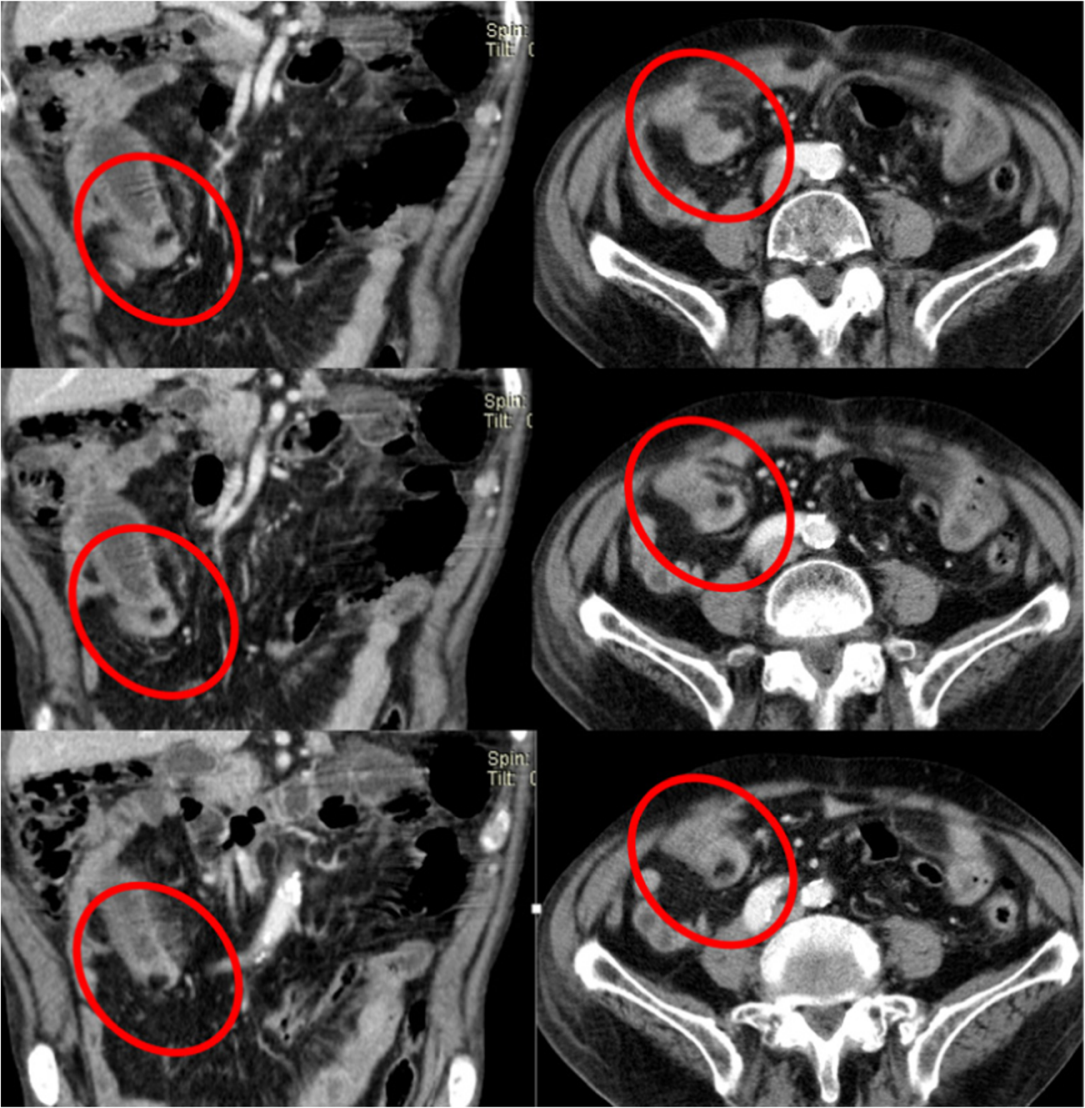
Patient with chronic abdominal pain and small-grade bowel obstruction. A fat density band is seen crossing the bowel loop in both the coronal and axial planes—fat-bridging sign (*circles*)

The diagnosis of small-bowel obstruction due to adhesions is presumed when all other causes of obstruction have been ruled out on CT. The main CT findings that suggest adhesions as the culprit of the obstruction include a narrow zone of transition without an identifiable lesion (such as a mass, wall thickening or adenopathy), acute angulation of the small-bowel loops, traction deformities, stretching of the bowel loops, small-bowel loops closely applied to the anterior abdominal wall and the “fat-bridging sign” which represents the adhesive band itself as a cord-like structure containing mesenteric fat that bridges two peritoneal surfaces [8, 9] (Figs. 10, 11, and 12).

Small-bowel obstruction caused by adhesions almost always requires surgery. When intestinal strangulation supervenes, immediate abdominal surgery is mandatory. However, the risk of additional adhesions increases as the number of surgeries increases.

### Afferent loop syndrome

Afferent loop syndrome (ALS) represents mechanical obstruction of the afferent loop, occurring in 0.3–2 % of gastroenterostomies, both in Billroth II surgery (the duodenum is the afferent loop) and Whipple or Roux-en-Y procedures (the Roux segment is the afferent loop) [9, 24, 25].

Most cases of ALS are due to adhesions, internal hernia, anastomotic stricture or recurrent tumor [9]. It can also be secondary to preferential gastric emptying into the afferent loop due to abnormal surgical anastomosis or because of efferent loop obstruction resulting in fluid accumulation at the afferent loop [24, 26]. The back pressure from the dilated afferent loop can cause biliary dilatation and acute pancreatitis [26].

The diagnosis may not be clinically suspected, as ALS may present many years after the initial surgery [27].

**Fig. 13:**
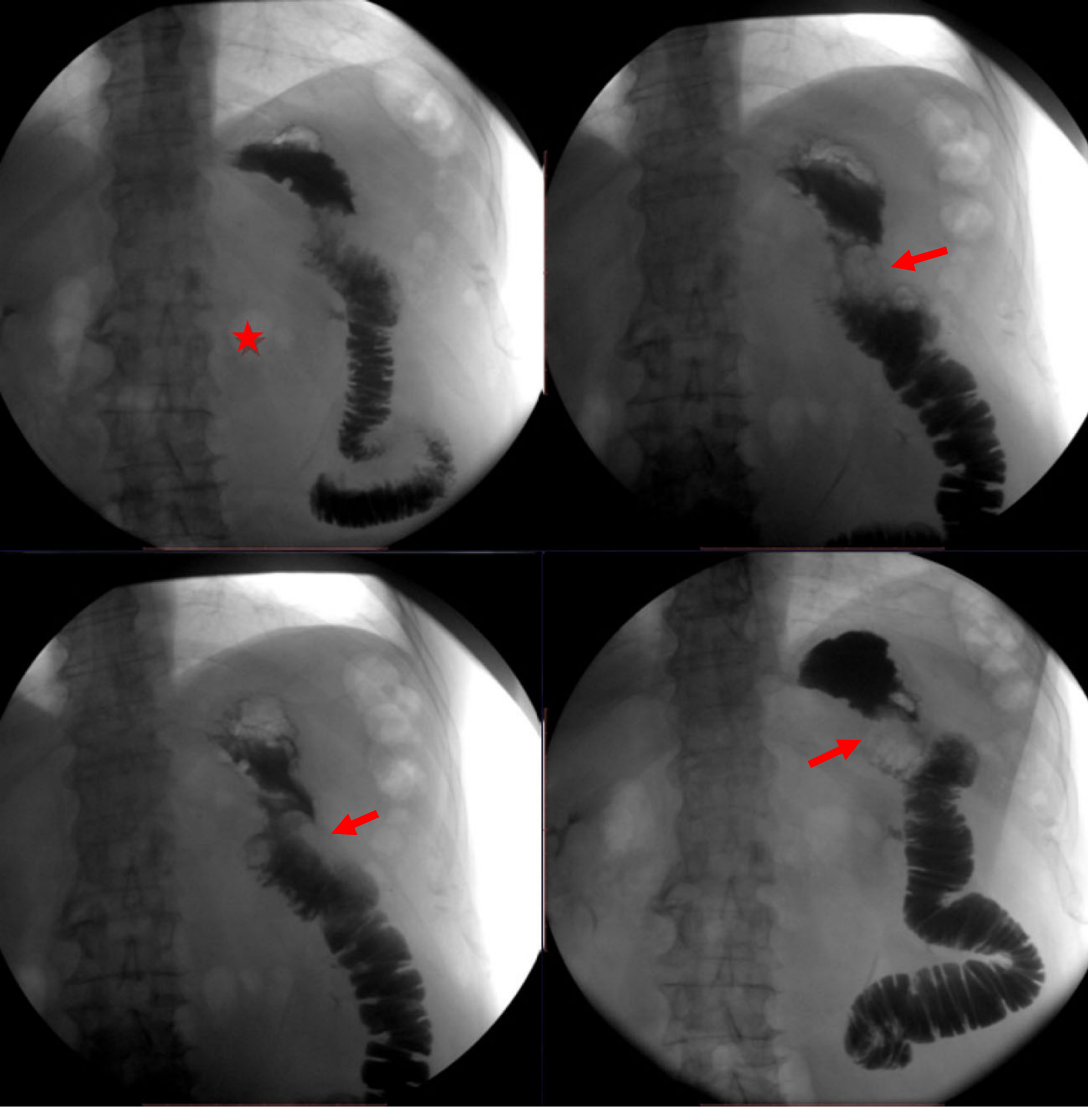
Upper gastrointestinal series of a patient with abdominal pain and vomiting who had undergone partial gastrectomy with Billroth II reconstruction following gastric adenocarcinoma 9 years earlier. There is non-filling of the afferent loop (*star*) and a filling defect at the location of the anastomosis (*arrows*)

Although gastrointestinal contrast studies can be helpful in the diagnosis of this condition by showing non-filling of the afferent loop, non-obstructed afferent loops are not normally filled in 20 % of cases. Preferential filling and retention of oral contrast in a dilated afferent limb for at least 60 min is another finding consistent with afferent loop syndrome [24, 26] (Fig. 13).

**Fig. 14:**
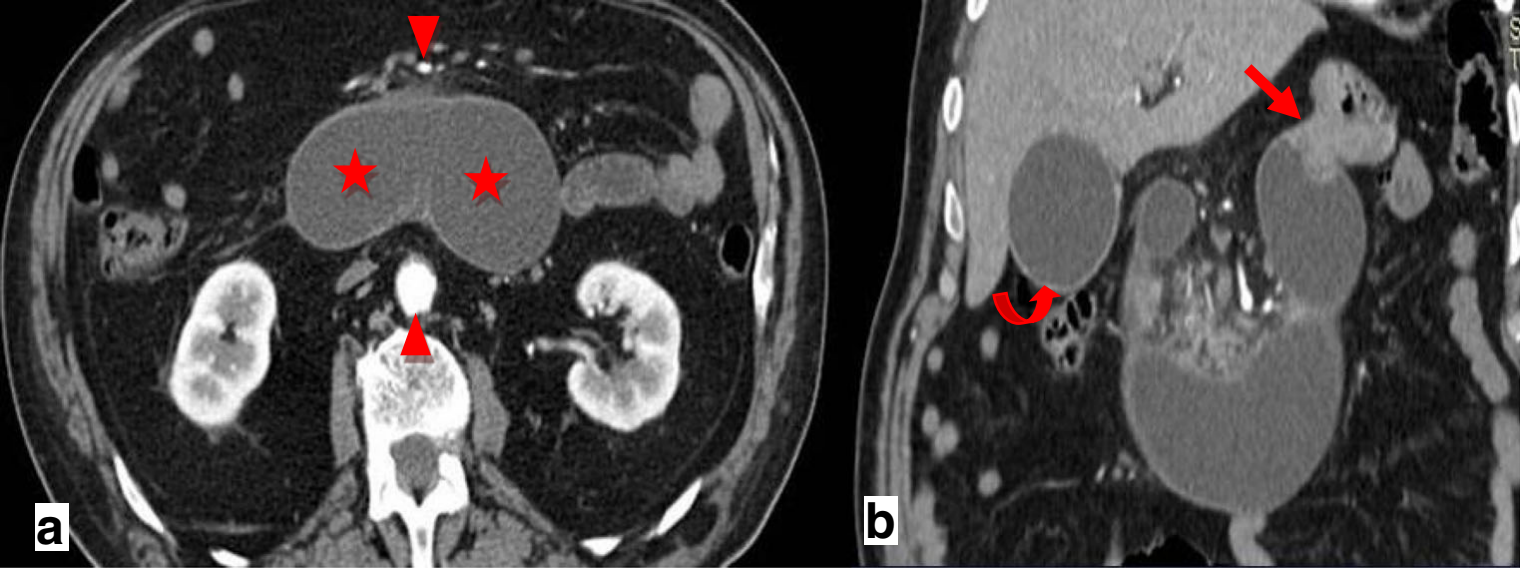
**a**, **b** Abdominal CT of the same patient as in Fig. 13. A fluid-filled tubular structure (*stars in a*) crossing the midline between the aorta and the superior mesenteric vessels (*arrowheads in a*) is seen, with associated gallbladder distension (curved arrow *in b*)–afferent-loop syndrome caused by tumor recurrence, depicted as an irregular enhancing mass at the anastomosis site (*arrow in b*)

**Fig. 15:**
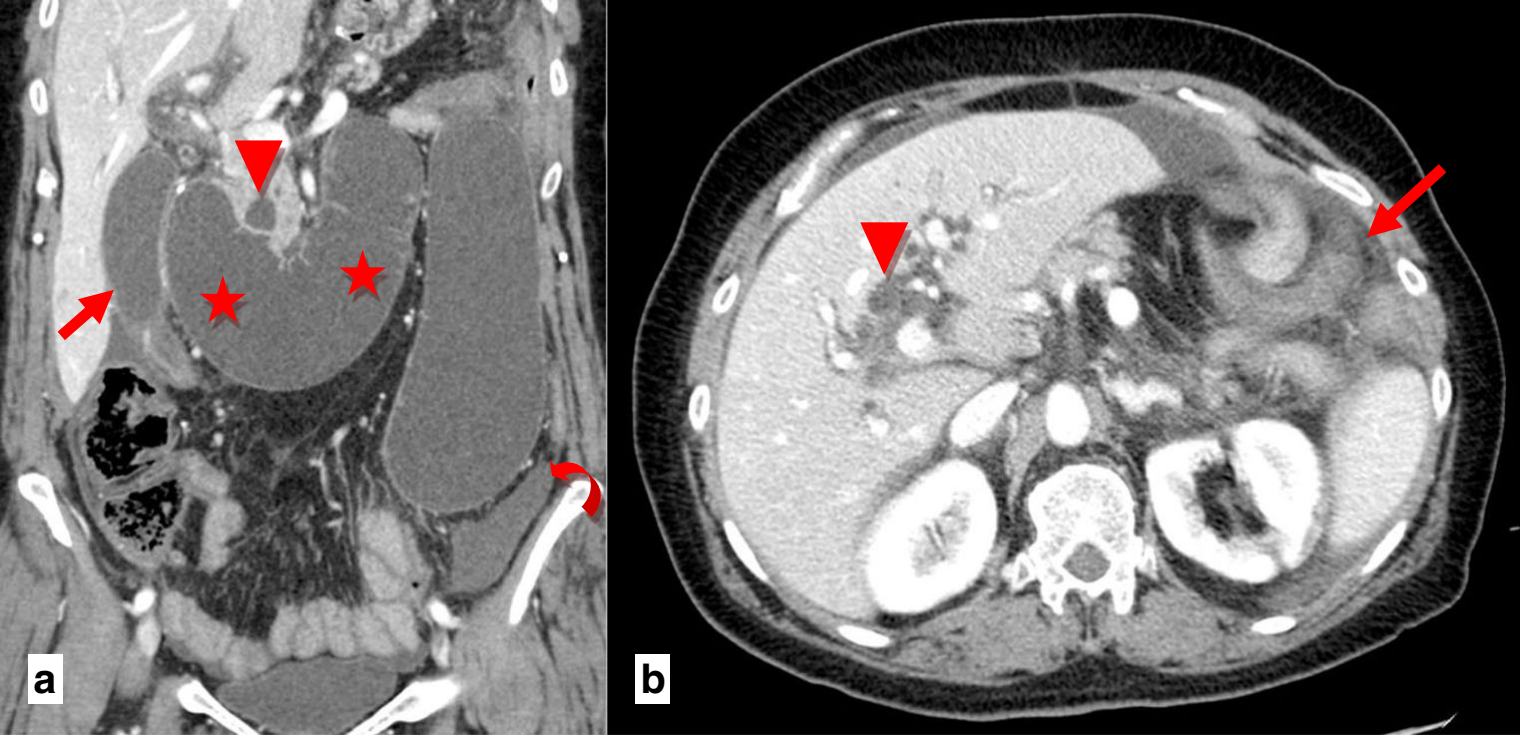
**a**, **b** Abdominal CT of a patient with progressively worsening abdominal pain, nausea and vomiting who had undergone Billroth II partial gastrectomy for gastric carcinoma 5 years earlier. There is marked fluid-filled dilation of the duodenum (*stars in a*), associated with gallbladder distension (*arrow in a*) and intrahepatic and extrahepatic biliary dilation (*arrowheads in a, b*), caused by an internal hernia with volvulus as shown by the twisted configuration of the bowel loops at the left upper quadrant (*arrow in b*). The ascites (*curved arrow in a*) suggests supervened ischemia

CT is the most important imaging tool in establishing the diagnosis and determining the site, degree and cause of ALS. A fluid-filled tubular or C-shaped structure containing small air bubbles in the right upper quadrant or crossing the midline between the aorta and the superior mesenteric vessels, with valvulae conniventes projecting into the lumen, in symptomatic patients after gastroenterostomy is characteristic (Fig. 14). Recognition of these distinctive findings should prevent the misdiagnosis of a pancreatic pseudocyst [24, 27]. The coronal plane is most helpful in detecting the causes of ALS so that adequate treatment can be chosen [24]. Complications of ALS such as biliary dilatation, pancreatitis or strangulation are also readily identified on CT [26] (Fig. 15).

### Anastomotic strictures

The incidence of anastomotic strictures depends on the type of surgery performed. The causes for the stricture are not yet clearly understood. For example, in gastric bypass patients, it is thought that the period of time during which the anastomosis is exposed to an inappropriately large volume of gastric acid results in ongoing inflammation, ulceration and stricture formation [28]. Several studies also suggest an increase in the incidence of strictures with the use of stapler versus handsewn anastomosis, both for gastrojejunostomy and colorectal anastomosis [1, 29]. For ileal pouch anal anastomosis, a meta-analysis found an incidence of anastomotic strictures of 9.2 % [30].

The resulting symptoms reflect the degree of luminal stenosis, and include abdominal pain, distention, nausea and vomiting.

**Fig. 16:**
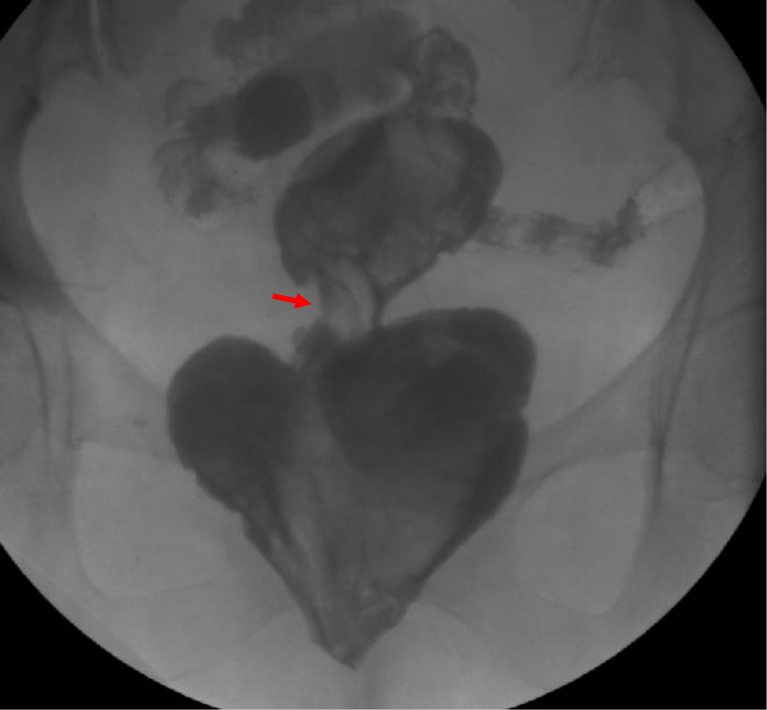
Water-soluble enema shows a short anastomotic stricture (*arrow*) and proximal dilatation in a patient who had undergone anterior resection of the rectum some months earlier

**Fig. 17:**
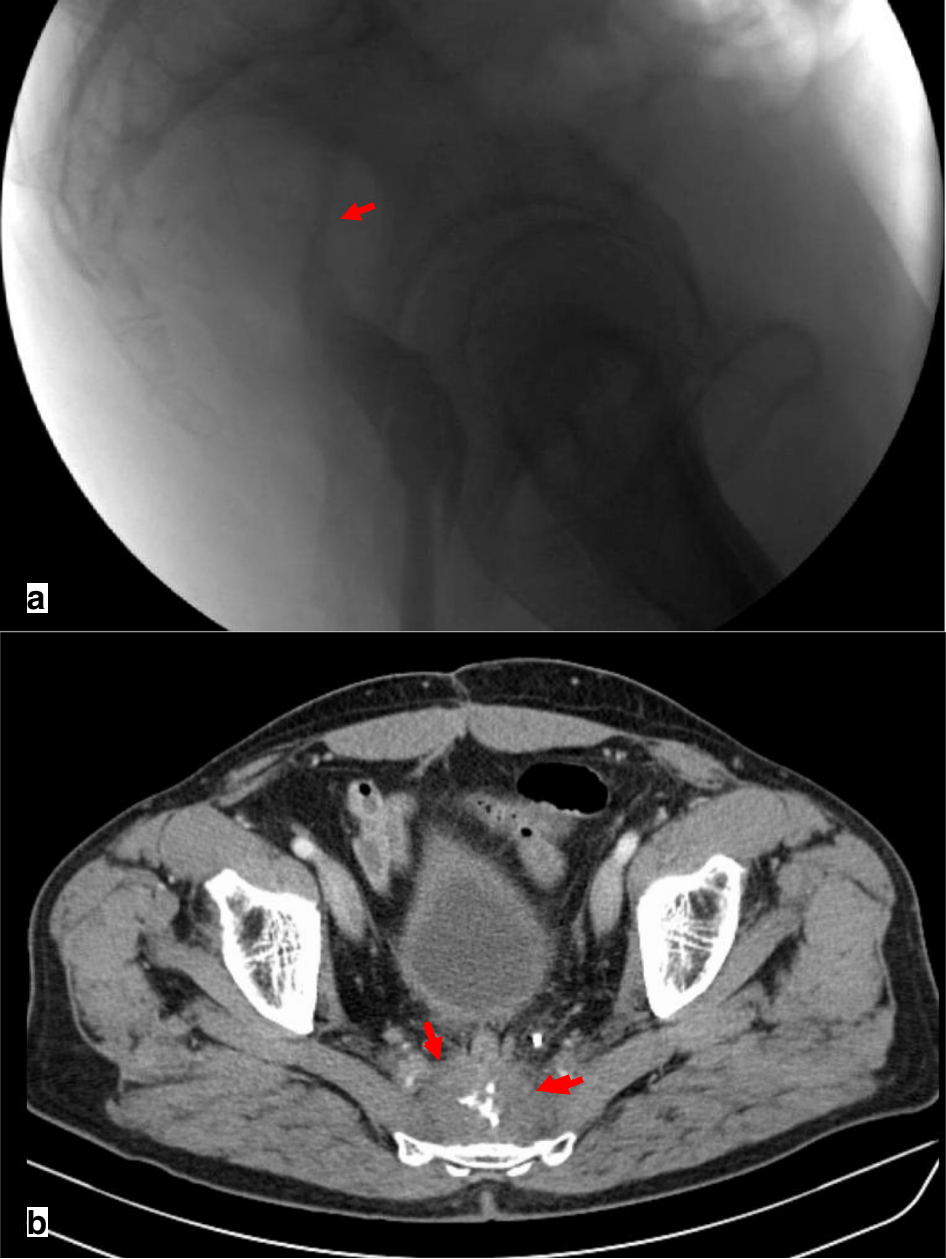
**a**, **b** Patient with complete lower intestinal obstruction who had undergone anterior resection of the rectum some years earlier. Water-soluble enema showed no progression of contrast beyond the coloanal anastomosis (*arrow in a*). Pelvic CT revealed diffuse wall thickening (*arrows in b*) of the coloanal anastomosis (note the surgical staples) due to a histologically proven fibrotic stenosis

Gastrointestinal contrast studies are the classical method for diagnosing anastomotic strictures. They allow a clear depiction of the stenosis with delayed passage of contrast material, pre-stenotic bowel distension and possible peri-anastomotic fistulae (Figs. 16 and 17a).

CT has become increasing important when an anastomotic stricture is being considered, and is now the preferred imaging modality in this particular setting, since it is able to demonstrate not only the anastomotic abnormality but extra-wall structures as well [1]. CT findings include focal bowel wall thickening at the anastomosis and distended proximal bowel loops filled with fluid or desiccated stool [31] (Fig. 17b).

Depending on the location, strictures can be treated with manual/endoscopic dilation or surgery [31].

## Disease-related complications

### Neoplastic recurrence

Tumor recurrence can be the cause of obstruction several months or years after surgery.

CT is the modality of choice in this setting, as it clearly shows an enhancing mass or asymmetric wall-thickening near the site of the initially resected tumor.

**Fig. 18:**
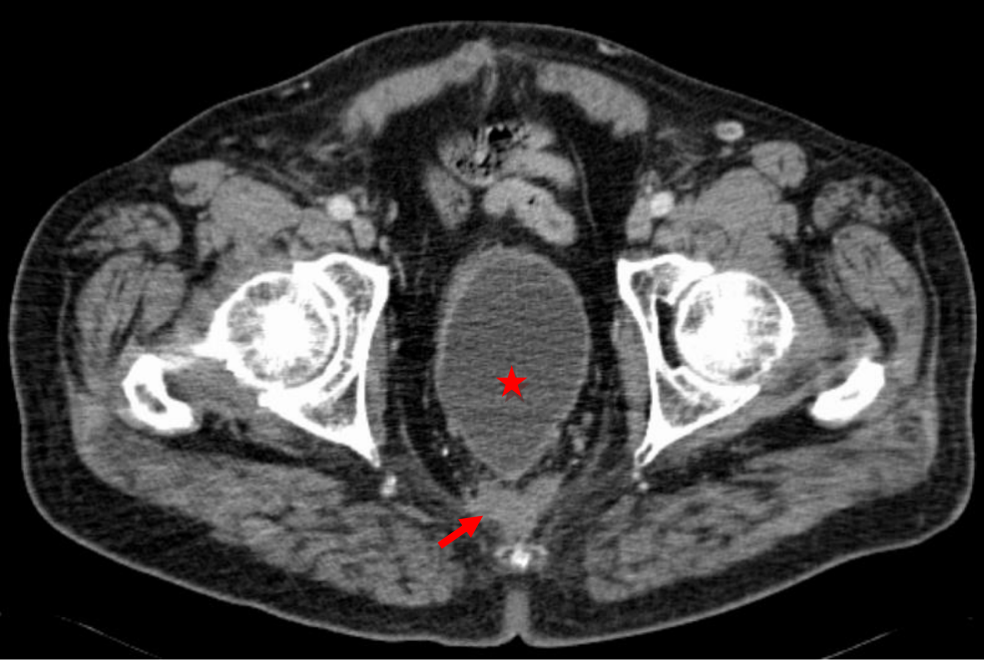
Pelvic CT scan of a patient who underwent abdominal perineal resection. Posteriorly to the bladder, there is an ill-defined irregular soft tissue mass corresponding to post-surgical fibrosis (*arrow*). Note the posterior displacement of the bladder (*star*) into the pre-sacral space

Most patients who undergo abdominal perineal resection or anterior resection of the rectum will present an ill-defined presacral midline mass 3 to 5 cm in diameter that decreases in size with time (although it can persist indefinitely) and becomes progressively more distinct in serial imaging—fibrosis / granulation tissue—a finding that can easily be mistaken for tumor recurrence [19] (Fig. 18).

**Fig. 19:**
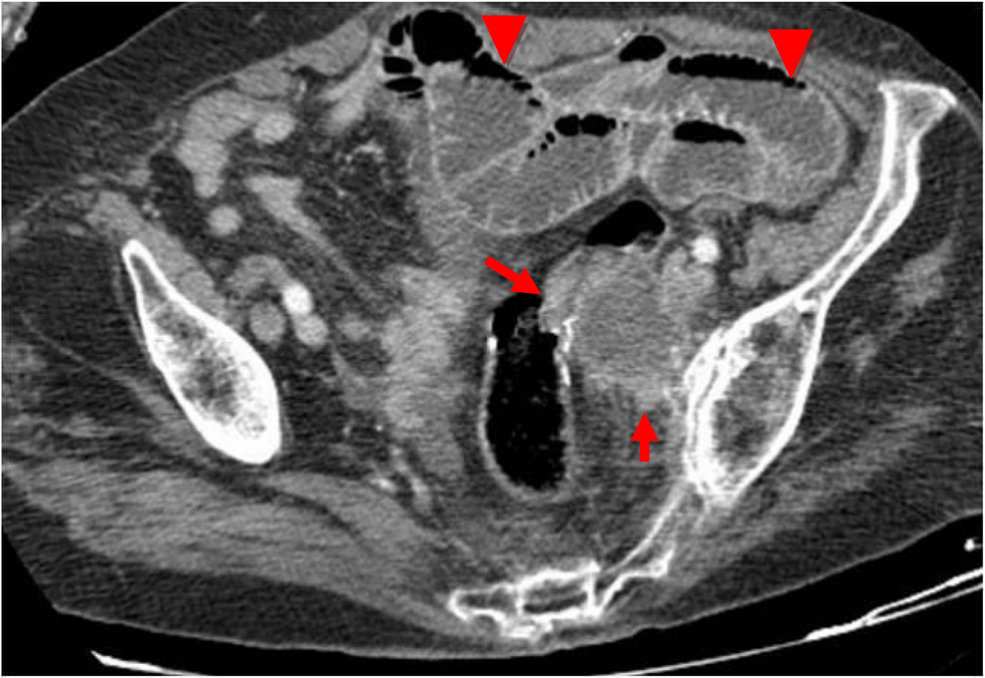
Contrast-enhanced pelvic CT of a 79-year old woman with obstructive symptoms who had undergone sigmoidectomy for adenocarcinoma 1 year earlier. There is asymmetric thickening of the bowel wall and a hypodense mass adjacent to the colorectal anastomosis (*arrows*), causing proximal bowel distension (*arrowheads*), confirmed as tumor recurrence at surgery

**Fig. 20:**
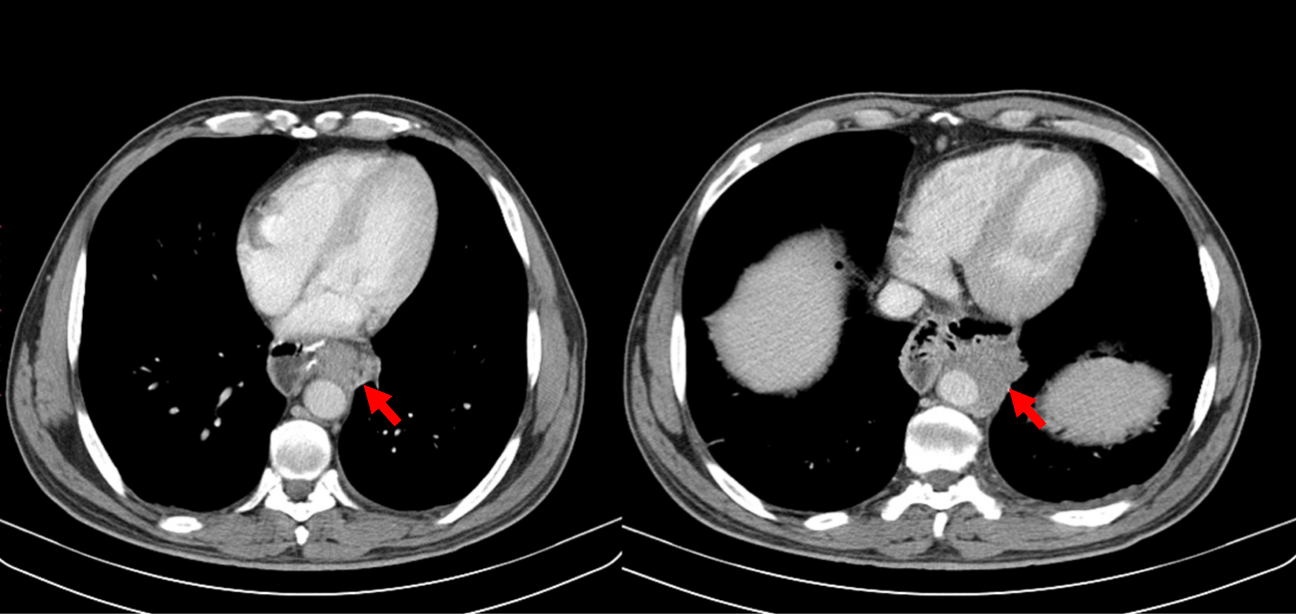
Thoracic-abdominal CT of a patient who had undergone total gastrectomy for adenocarcinoma some years earlier. There is evidence of a soft tissue mass next to the surgical anastomosis, encircling the descending aorta (*arrows*) and corresponding to a non-resectable neoplastic recurrence

In contrast, tumor recurrence manifests as a well-defined soft tissue mass that grows on serial imaging and becomes ill-defined as it becomes more infiltrative [19] (Figs. 19 and 20).

### Inflammatory bowel disease recurrence

#### Crohn’s disease

Most patients with Crohn’s disease (CD) require surgery at some point in their lives, and can develop relapses either at the intestinal anastomosis or far from it, at any other segment of the GI tract. These relapses can occur in the form of new strictures, fistulae or abscesses [1].

A combination of symptom assessment plus endoscopy is still the gold standard for assessment of recurrence in postoperative CD patients [32].

CT enterography/enteroclysis should be the first radiologic procedure performed in patients with acute symptoms when recurrence is suspected. MR enterography/enteroclysis is increasingly being considered as the first-choice examination in acute exacerbation in a child or young adult with known CD, because it is a radiation-sparing technique in a patient who will likely be subjected to multiple serial examinations [33]. MRI is also the technique of choice for the diagnosis and characterization of perianal fistulae.

**Fig. 21:**
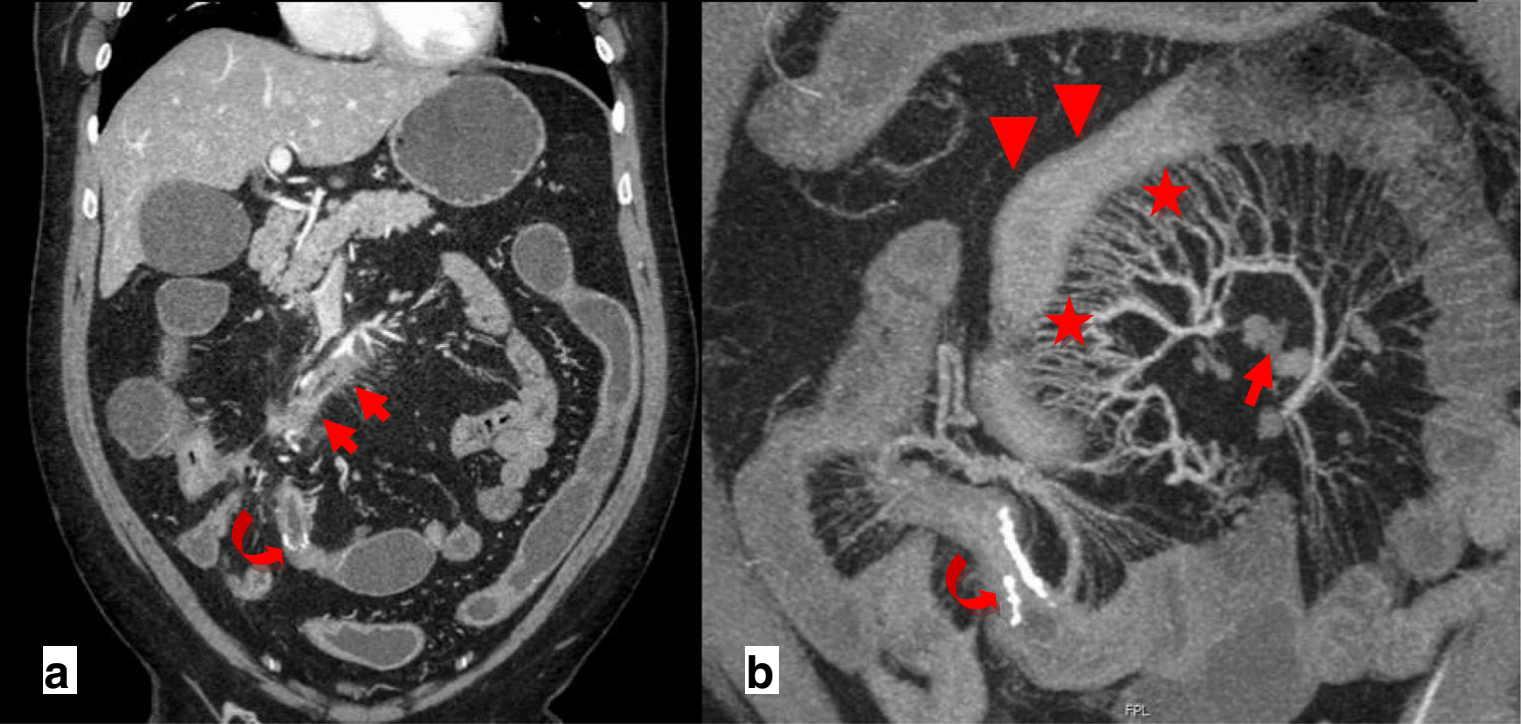
**a**, **b** CT enterography of a patient with Crohn’s disease who had previously undergone segmental enterectomy. Note the surgical material at the ileal-ileal anastomosis (*curved arrows in a, b*). There is a fistulous tract between the thickened ileal segment near the anastomosis and the duodenum (*arrows in a*). MIP coronal reformation (*b*) shows mesenteric engorgement (*stars in b*) and lymphadenopathy (*arrow in b*) in the vicinity of a thickened bowel wall segment (*arrowheads in b*)

**Fig. 22:**
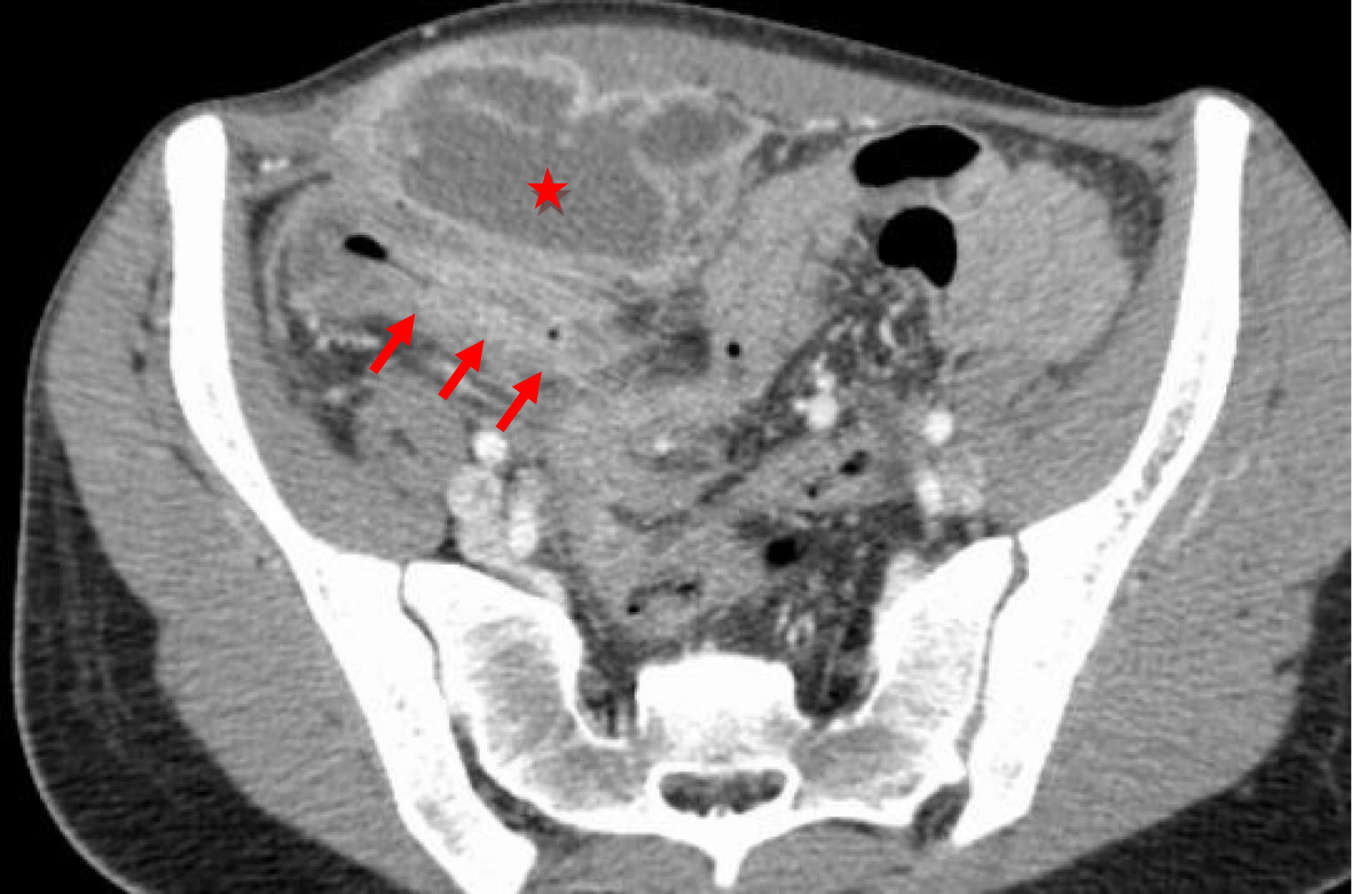
A fluid collection with a thickened, hyper-enhancing wall corresponding to an abscess (*star*) is seen anteriorly to a bowel wall segment with thick walls at the level of the neo-terminal ileum (*arrows*) in a patient who had already undergone surgery due to Crohn’s terminal ileitis

The ability to directly demonstrate the bowel wall, adjacent organs, mesentery and retroperitoneum renders CT and MR superior to any other examination in diagnosing the complications of CD. Both methods can evidence hyper-enhancing bowel wall thickening, mesenteric edema, prominent mesenteric vessels (the “comb sign”) and lymphadenopathy, as well as possible fistulae or abscesses (Figs. 21 and 22).

**Fig. 23:**
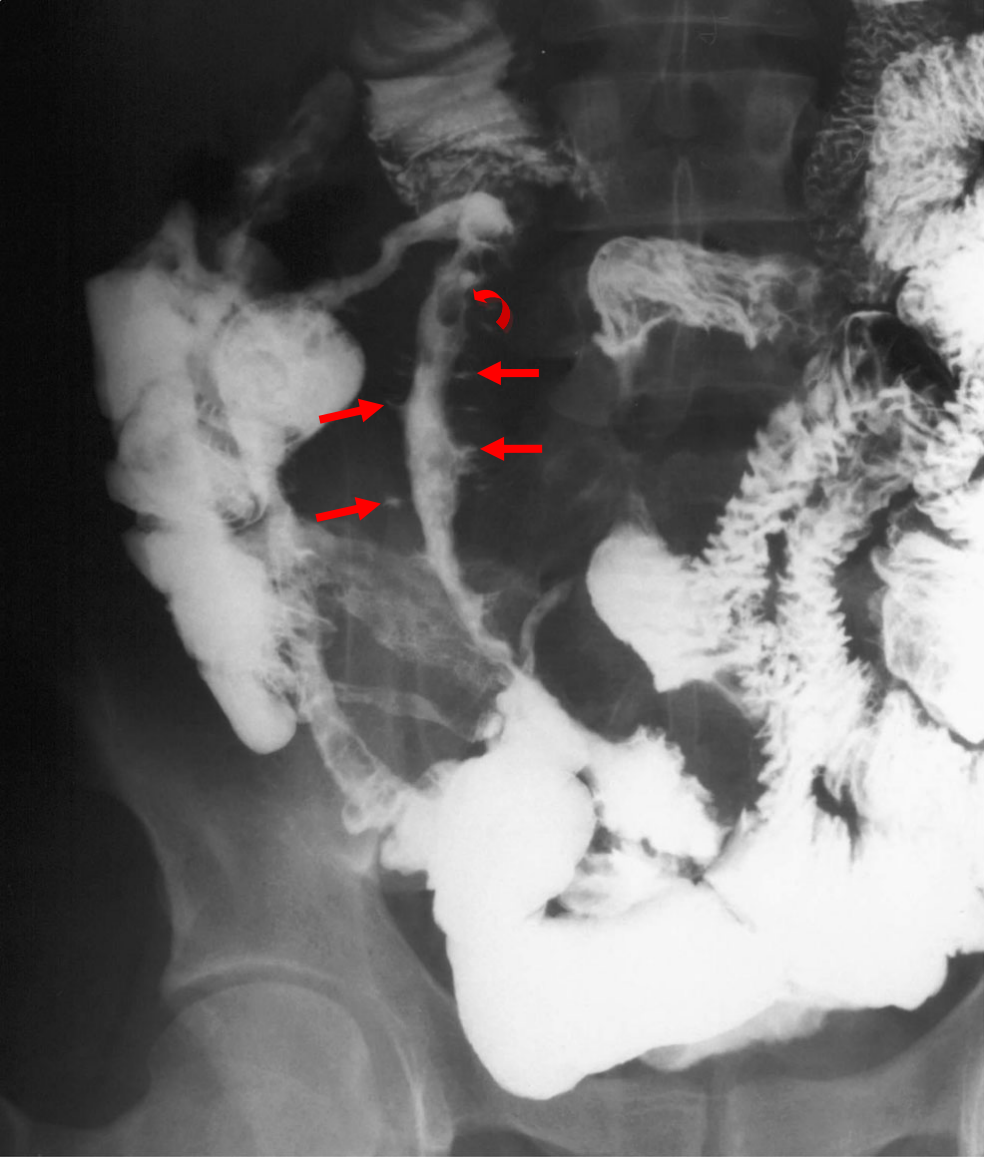
Barium follow-through of a patient with active Crohn’s disease: there is severe irregular narrowing of the neo-terminal ileum, with separation from adjacent bowel loops and with some areas of cobblestoning (*curved arrow*) and linear deep ulcers (*arrows*)

Although CT is by far the most frequent examination undertaken in this setting, we cannot simply discard gastrointestinal contrast studies just yet. A recent study found small bowel follow-through (SBFT) to be more sensitive and specific than CT for detecting recurrent CD in the neo-terminal ileum because of its ability to depict aphthoid ulcers not detectable on CT. Other findings of Crohn’s recurrence at SBFT are luminal narrowing, thickened folds and deep ulcerations (“rose thorn” ulcers) [34] (Fig. 23).

#### Ulcerative colitis

Ulcerative colitis is normally continuous from the rectum to the colon. Total proctocolectomy is the definitive treatment for the disease, but two other types of surgery are usually performed to obviate the disadvantages of a permanent colostomy.

Patients who receive total colectomy with ileorectal anastomosis are at risk for disease recurrence at the rectum, and thus clinical and endoscopic follow-up are advised. When proctocolectomy with ileal-anal pouch is chosen, there is an increased probability of the patient developing pouchitis (ileal pouch inflammation). The diagnosis depends on clinical, endoscopic and histopathological data, and there is usually no role for diagnostic imaging in this setting [1].

## Conclusions

In conclusion, knowledge of the normal appearance of the abdomen and pelvis after GI tract surgery is mandatory for every diagnostic radiologist, particularly in an emergency setting. An acquaintance with the most frequent complications in the late postoperative period, such as bowel obstruction due to internal or external hernias, adhesions, afferent loop syndrome and disease recurrence, is of paramount importance.

Communication with the referring surgeon can be a valuable adjunct for achieving diagnosis in potentially life-threatening situations so that adequate therapy can be planned.
